# Effects of Hydrogen Peroxide and Ultrasound on Biomass Reduction and Toxin Release in the Cyanobacterium, *Microcystis aeruginosa*

**DOI:** 10.3390/toxins6123260

**Published:** 2014-12-10

**Authors:** Miquel Lürling, Debin Meng, Elisabeth J. Faassen

**Affiliations:** 1 Aquatic Ecology and Water Quality Management Group, Department of Environmental Sciences, Wageningen University, P.O. Box 47, 6700 AA Wageningen, The Netherlands; E-Mails: mengdebin815@hotmail.com (D.M.); els.faassen@wur.nl (E.J.F.); 2 Department of Aquatic Ecology, Netherlands Institute of Ecology (NIOO-KNAW), P.O. Box 50, 6700 AB Wageningen, The Netherlands

**Keywords:** cyanotoxin, eutrophication control, lake restoration, LC-MS/MS, microcystin profile, PCC 7820

## Abstract

Cyanobacterial blooms are expected to increase, and the toxins they produce threaten human health and impair ecosystem services. The reduction of the nutrient load of surface waters is the preferred way to prevent these blooms; however, this is not always feasible. Quick curative measures are therefore preferred in some cases. Two of these proposed measures, peroxide and ultrasound, were tested for their efficiency in reducing cyanobacterial biomass and potential release of cyanotoxins. Hereto, laboratory assays with a microcystin (MC)-producing cyanobacterium (*Microcystis aeruginosa*) were conducted. Peroxide effectively reduced *M. aeruginosa* biomass when dosed at 4 or 8 mg L^−1^, but not at 1 and 2 mg L^−1^. Peroxide dosed at 4 or 8 mg L^−1^ lowered total MC concentrations by 23%, yet led to a significant release of MCs into the water. Dissolved MC concentrations were nine-times (4 mg L^−1^) and 12-times (8 mg L^−1^ H_2_O_2_) higher than in the control. Cell lysis moreover increased the proportion of the dissolved hydrophobic variants, MC-LW and MC-LF (where L = Leucine, W = tryptophan, F = phenylalanine). Ultrasound treatment with commercial transducers sold for clearing ponds and lakes only caused minimal growth inhibition and some release of MCs into the water. Commercial ultrasound transducers are therefore ineffective at controlling cyanobacteria.

## 1. Introduction

Cyanobacterial proliferations and the formation of surface scums are among the most noticeable and malignant consequences of eutrophication [1]. Cyanobacterial blooms are a serious water quality threat, as blooms may produce nasty odors, cause high turbidity, anoxia, fish kills and food web alterations [2,3]. Furthermore, because cyanobacteria can produce a variety of potent toxins [4], cyanobacterial blooms exert strong pressure on important ecosystems services, such as recreation, aquaculture, irrigation and drinking water preparation. As a consequence, cyanobacterial blooms have severe economic impacts [5]. 

Because cyanobacterial blooms can deteriorate the water quality below the level that is needed for drinking water production, irrigation, industry, recreation and fishing, water managers try to control the massive development of cyanobacterial biomass. Nutrient reduction in the water body and its catchment area is clearly the most prominent effective approach to prevent cyanobacterial dominance [6]. However, reduction to sufficiently low nutrients loads may take fairly long to realize, and sometimes, it may not be feasible at all. Hence, in systems where the reduction of external nutrient loading is not economically feasible, effect-oriented (curative) measures may provide the most suitable nuisance control [7]. Such applications should be fast-acting and strongly reduce cyanobacteria biomass, so that safe water quality levels are rapidly reached [7]. In decision making for mitigating measures besides biomass also the production of cyanobacterial toxins are increasingly being taken into account [8], as these cyanotoxins make cyanobacterial blooms and surface scums a threat to environmental health and public safety [9,10]. While reducing biomass, care should be taken that the cyanobacterial toxins are not released into the water. Most cyanobacterial toxins are largely contained within the cyanobacterial cells, until lysis or damage of the cells liberates them [11,12]. Curative measures that result in the release of toxins from the cells, like the application of copper [13], endanger rather than improve water quality [4]. 

There are several promising curative measures to control cyanobacterial biomass. Copper is among the most applied algaecides [7], but toxicity issues using copper [4] have led to the promotion of hydrogen peroxide (H_2_O_2_) as an effective non-toxic alternative [14,15]. Hydrogen peroxide is a powerful oxidizing agent that acts via the formation of hydroxyl radicals (·OH), which oxidize thiol groups in biomolecules [16]. However, it has been stated that the use of algaecides should be avoided, since it may lead to significant toxin release [4]. This finds some support in a whole lake experiment, in which both cyanobacteria biomass and the cyanotoxin microcystin (MC) strongly declined after application with H_2_O_2_, albeit with a two-days’ time delay for MC [15], which might point towards a shift from particulate to dissolved MC and, thus, cyanotoxin release. A different technique that has been labelled as an “environmental friendly” or “green solution” to kill cyanobacteria is the use of ultrasound [17,18]. Nevertheless, a vast majority of studies on this subject have been performed with high power devices designed for cleaning and sterilizing that differ significantly from those sold on the market for controlling phytoplankton in lakes and ponds [19]. High power devices could lead to a strong increase in extracellular MC concentrations [20], but could also cause MC degradation [21,22] through peroxide formation and hydroxyl radicals [22]. The effect of commercially available ultrasound transducers on cyanobacterial toxin release is unknown. Thus, while both peroxide and ultrasound have been reported as promising measures in reducing cyanobacterial biomass, they consequently might cause a release of cyanotoxins. 

**Figure 1:**
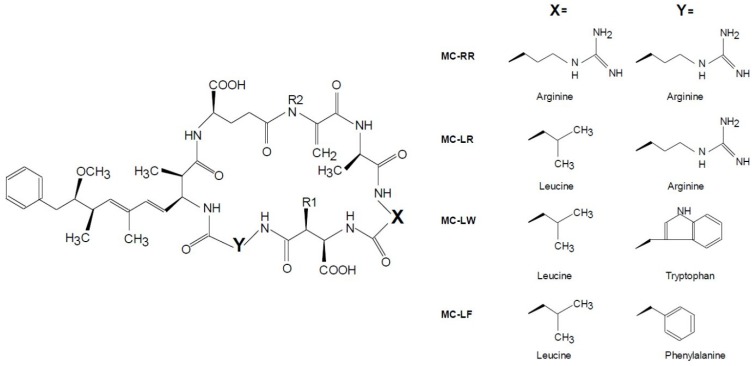
General structure of microcystins and examples of substitutions at position *X* (L = leucine, R = arginine) and *Y* (R = arginine, W= tryptophan, F = phenylalanine) resulting in the variants microcystin MC-RR, MC-LR, MC-LW and MC-LF, if positions R1 and R2 are methylated.

Microcystins (MCs) are the most frequently occurring cyanotoxins. MCs are produced by a diverse range of cyanobacteria, of which *Microcystis* is one of the most common bloom formers [23,24,25]. MCs are non-ribosomal processed cyclic heptapeptides with a size between 909 Da and 1115 Da [26]. The general structure is cyclo(-d-ala-l-*X*-erythro-β-d-methylaspartic acid-l-*Z*-Adda-d-isoglutamic acid-*N*-methyldehydroalanine), where Adda is (2S,3S,8S,9S)-3-amino-9-methoxy-2,6,8-trimethyl-10-phenyldeca-4,6-dienoic acid [27] (Figure 1). *X* and *Z* are variable l-amino acids contributing mostly to the dozens of variants of MCs that have been detected [27]. MCs are potent inhibitors of protein phosphatases, but the toxicity of different variants to mice varies substantially, where replacement of the hydrophobic leucine (L) in the first variable position with a hydrophilic amino acid (e.g., arginine, R) dramatically reduces toxicity [28] (Figure 1).

Little is known about how these different MC variants react to curative measures to control cyanobacterial biomass. The degradation rate of two MC variants (MC-LR and MC-RR, with L = Leucine and R = Arginine) upon irradiation with high power ultrasound (500 W, applied to a 22-mL reaction vessel) differed with an estimated EC_50_ of 30 min for MC-RR and 70 min for MC-LR [21]. However, the effect of commercially available ultrasound transducers on different MC variants has not been examined yet. Likewise, the few studies on H_2_O_2_ control of cyanobacteria that included MC analysis [15,29,30] did not specify the effects on different MC variants. Therefore, in this study, we tested the hypothesis that both H_2_O_2_ treatment and ultrasound from commercial transducers sold for clearing lakes are effective at strongly reducing cyanobacteria biomass, without increasing the MC concentration in the water. Furthermore, we hypothesized that all measured MC variants reacted in a similar manner to these two treatments. Hereto, we ran laboratory experiments with a *M. aeruginosa* strain that, amongst others, produces the five MC variants: dm-7-MC-LR, MC-LR, MC-LY, MC-LF and MC-LW (dm = demethylated at position R2 in Figure 1: R2 = H).

## 2. Results 

### 2.1. Hydrogen Peroxide

**Figure 2:**
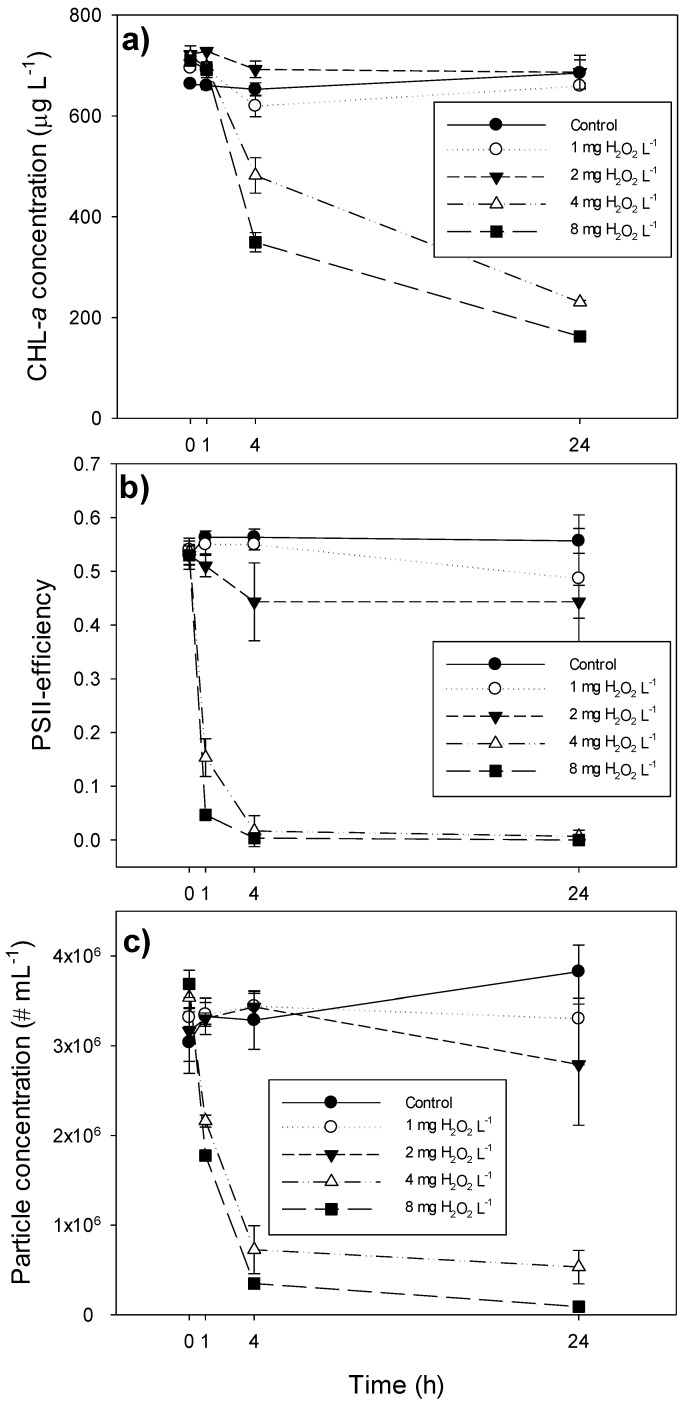
(**a**) Chlorophyll-*a* concentrations (CHL-*a*, μg L^−1^); (**b**) photosystem II efficiency (PSII); and (**c**) particle concentration (No. mL^−1^) of *Microcystis aeruginosa* PCC 7820 exposed to different H_2_O_2_ concentrations for 24 h. Error bars indicate 1 SD (*n* = 3).

H_2_O_2_ application of 4 and 8 mg L^−1^ significantly lowered the cyanobacterial chlorophyll-*a* and particle concentration after 24 h (Figure 2; Table 1). The chlorophyll-*a* concentration was reduced to approximately 200 µg L^−1^ in the highest H_2_O_2_ treatments (Figure 2a), but the particle concentration was reduced to ~9 × 10^4^ particles mL^−1^ after 24 h in the 8-mg L^−1^ H_2_O_2_ treatment (Figure 2c). The photosynthetic efficiency of the cyanobacteria, expressed as photosystem II efficiency (Φ_PSII_), was more sensitive to H_2_O_2_ application; it was slightly reduced at 1 and 2 mg L^−1^, but became (virtually) zero at 4 and 8 mg L^−1^ (Figure 2b; Table 1). The H_2_O_2_ concentration at which 50% of the cyanobacteria were affected (EC_50_) ranged from 2.5 mg L^−1^ for the photosynthetic efficiency, to 3.8 mg L^−1^ for cyanobacterial chlorophyll-*a* (Table 1).

**Table 1:** Statistical information on the H_2_O_2_ experiment for three different endpoints: chlorophyll-*a* concentration (Chl-*a*), photosystem II efficiency (Φ_PSII_) and particle concentration (# mL^−1^) of *Microcystis aeruginosa* PCC 7820. Homogeneous subgroups were defined by a Tukey *post hoc* comparison at *p* < 0.05 and are indicated by similar symbols (a,b,c) per column.

H_2_O_2_ treatment	Chl-*a* (µg L^−1^)		Φ_PSII_ (-)		Particles (No. mL^−1^)	
	Tukey *post hoc* comparison					
Control	a		a		a	
1 mg L^−1^	a		a,b		a	
2 mg L^−1^	a		b		a	
4 mg L^−1^	b		c		b	
8 mg L^−1^	c		c		b	
repeated measures ANOVA	*F*	*p*	*F*	*p*	*F*	*p*
treatment effect (*F*_4,10_)	367.5	<0.001	284.1	<0.001	102.4	<0.001
time effect (*F*_3,30_)	695.3	<0.001	145.5	<0.001	80.0	<0.001
time × treatment interaction (*F*_12,30_)	215.0	<0.001	46.5	<0.001	41.1	<0.001
nonlinear regression		*r*^2^ adj		*r*^2^ adj		*r*^2^ adj
EC_50_ (mg H_2_O_2_ L^−1^)	3.8	0.992	2.5	0.947	2.6	0.929

**Figure 3:**
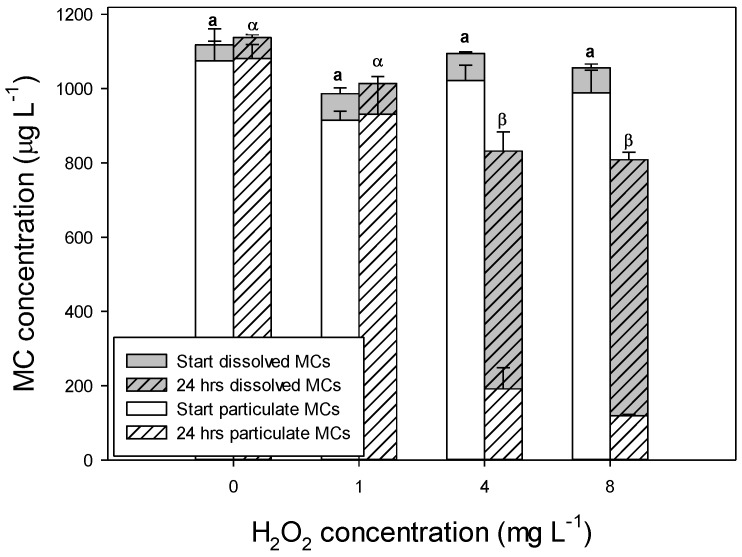
Total particulate and dissolved microcystin (MC) concentrations (μg L^−1^) in *Microcystis aeruginosa* PCC 7820 at the start of the experiment (open bars) and after 24 h of exposure to different concentrations of hydrogen-peroxide (H_2_O_2_, in mg L^−1^; hatched bars). Error bars indicate 1 SD (*n* = 3). Similar symbols above the bars (a,α,β) indicate homogeneous groups of total MC concentrations that are not different at the *p* = 0.05 level (Holm–Sidak comparison).

MCs were determined at the start and after 24 h in the controls and the 1-, 4- and 8-mg H_2_O_2_ L^−1^ treatments (Figure 3). Total MC concentrations (sum of dissolved and particulate MCs) were similar at the start of the experiment (one-way ANOVA, *F*_3,11_ = 3.23; *p* = 0.082), but after 24 h, the total MC concentrations were lower in the H_2_O_2_-treated jars than in the controls (*F*_3,11_ = 13.5; *p* = 0.002). The total MC concentrations in the highest H_2_O_2_ treatments had dropped 23 to 24% compared to their initial values (Figure 3).

At the start of the experiment, on average, 6.0% (SD 1.6%) of the total MC pool consisted of dissolved MCs. After 24 h, a similar percentage (6.5%, SD 1.9%) was found for the controls and the 1-mg H_2_O_2_ L^−1^ treatment, whereas in the 4- and 8-mg L^−1^ treatments, the proportion of dissolved MCs had increased to 77% (4 mg L^−1^) and 85% (8 mg L^−1^; Figure 3). H_2_O_2_ application significantly increased the dissolved MC concentrations (one-way ANOVA; *F*_3,11_ = 408.9; *p* < 0.001): dissolved MC concentrations in the controls and the 1-mg H_2_O_2_ L^−1^ treatment were significantly lower than those in the 4- and 8-mg H_2_O_2_ L^−1^ treatments (Holm–Sidak comparison, *p* < 0.05). 

**Figure 4:**
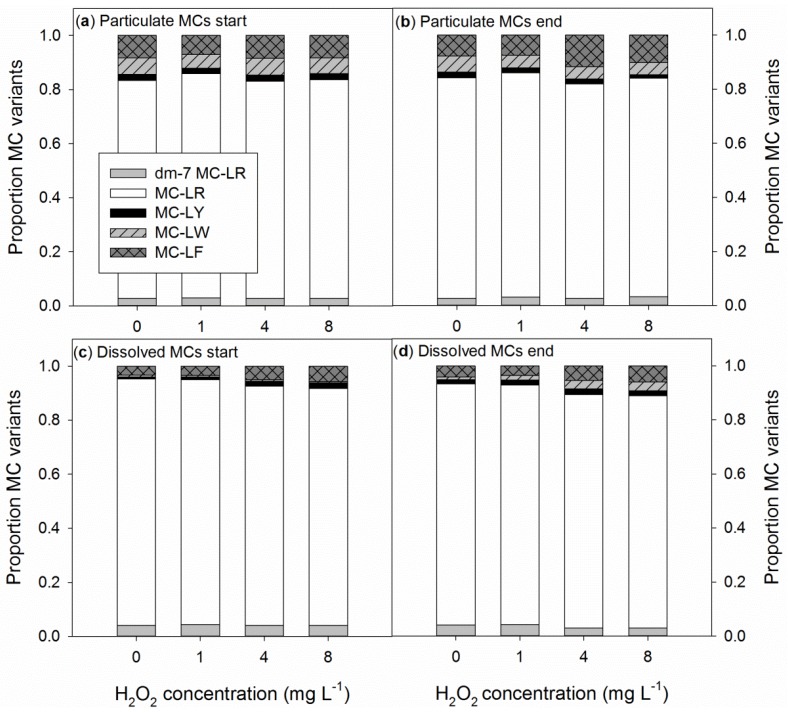
Proportions of five microcystin (MC) variants in the particulate (**a**,**b**); and dissolved MC pools (**c**,**d**) of *Microcystis aeruginosa* PCC 7820 at the start of the experiment (start) and after 24 h of exposure to different concentrations of hydrogen-peroxide (H_2_O_2_, in mg L^−1^; end).

The composition of particulate MCs did not differ much during the experiment and between treatments (Figure 4a,b). MC-LR was the most abundant MC variant, making up on average 81% of the particulate MCs; MC-LF was present at 9%, MC-LW at 5% and dm-7-MC-LR at 3%, and MC-LY was the least abundant variant with 2% abundance (Figure 4a,b). The composition of the dissolved MCs at the start of the experiment deviated from the composition of particulate MCs (Figure 4a,c). This effect was strongest for the hydrophobic variant, MC-LW, which formed more than 5% of the particulate MCs, but only made up 0.6% of the dissolved MC pool at the start of the experiment (two-way ANOVA; *F*_1,23_ = 969.3; *p* < 0.001). Furthermore, the contribution of MC-LF, another hydrophobic variant, to the overall dissolved MC pool was reduced by half (to 4%) compared to the particulate pool (Figure 4a,c). Here, two-way ANOVA not only indicated a significant difference between the contribution of dissolved MC-LF and particulate MC-LF to the overall MC pool (*F*_1,23_ = 969.3; *p* < 0.001), but also revealed that in the dissolved pool, differences already appeared at the start of the experiment (*F*_3,23_ = 11.0; *p* < 0.001) with dissolved MC-LF contributions of 3% in the control and the 1-mg L^−1^ treatment and 5% in the 4- and 8-mg L^−1^ treatments (Figure 4c). At the end of the experiment, the proportion of dissolved MC-LW increased, particularly in the 4- and 8-mg L^−1^ H_2_O_2_ treatments. Furthermore, the proportion of dissolved MC-LF increased slightly in these treatments, at the expense of the proportion of the more hydrophilic MC-LR (Figure 4d). As a consequence, the dissolved MC profiles in the highest H_2_O_2_ treatments resembled the particulate MC profiles more than did the dissolved MC profiles of the control and the 1-mg L^−1^ treatment (Figure 4). Because MC-LW and MC-LF likely contribute more to the total MC toxicity than MC-LR, the dissolved MC pool of lysed cells was relatively more toxic than the dissolved pool of healthy cells.

### 2.2. Ultrasound

**Figure 5:**
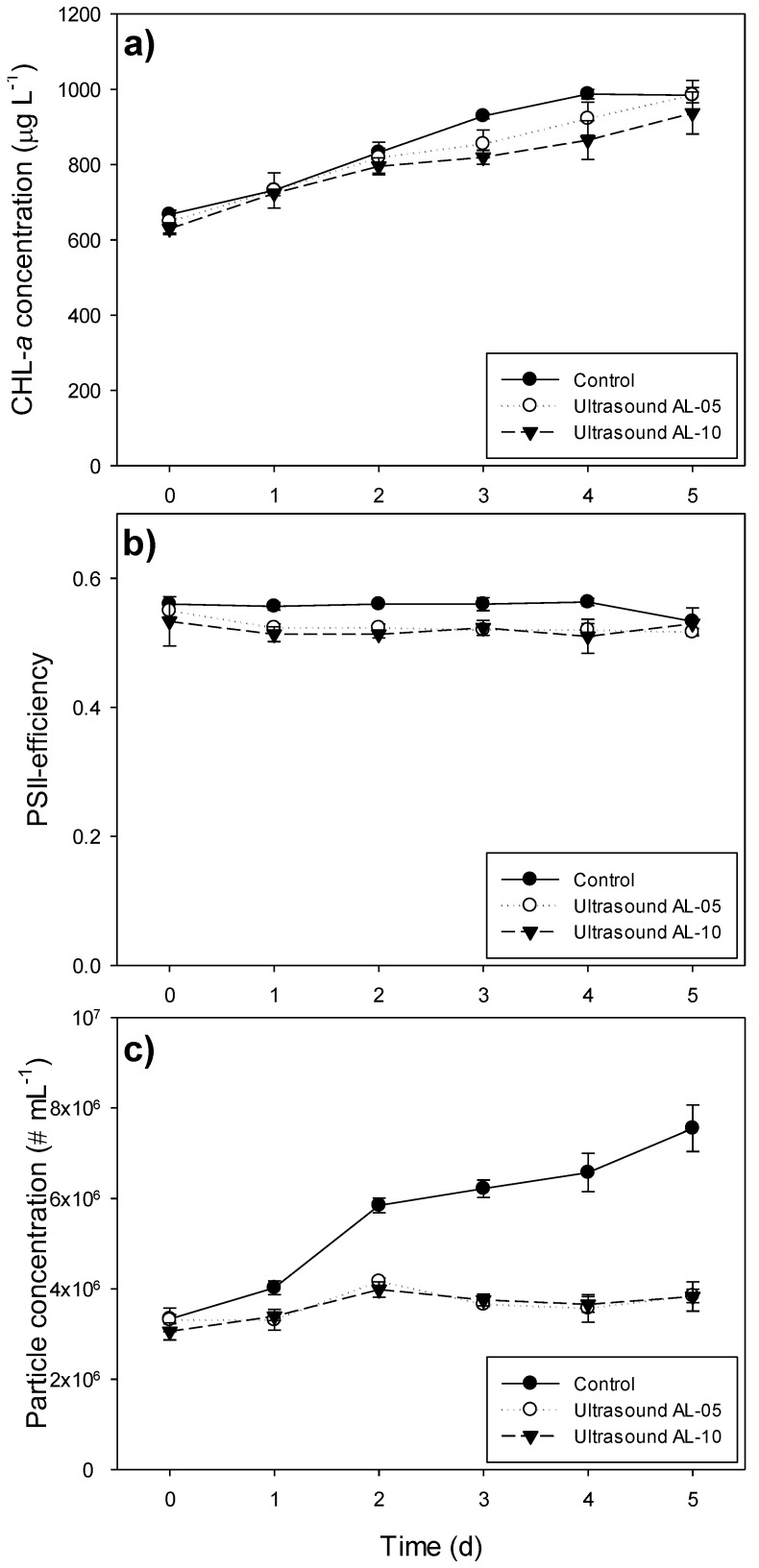
(**a**) Chlorophyll-*a* concentrations (CHL-*a*, μg L^−1^); (**b**) photosystem II efficiency (PSII); and (**c**) particle concentration (No. mL^−1^) in *Microcystis aeruginosa* PCC 7820 exposed to different ultrasound transducers (Flexidal AL-05 and AL-10) and in non-exposed control populations (control). Error bars indicate 1 SD (*n* = 3).

Despite the application of ultrasound, chlorophyll-*a* concentrations in both ultrasound treatments increased as strongly as the control treatment during the five-day course of the experiment (Figure 5; Table 2). The particle concentration, however, increased more in the control (2.3-times) than in the two ultrasound treatments (1.2-times; Figure 5c; Table 2). The Φ_PSII_ was slightly, but significantly, reduced by the ultrasound treatments, from an average of 0.56 (SD 0.01) in the controls during the five-day exposure period, to 0.53 (SD 0.01) in the AL-05 treatments and 0.52 (SD 0.02) in the AL-10 treatments (Figure 5b; Table 2). This effect was significant, because the within group variability was very low (Figure 5b).

**Table 2:** Statistical information on the ultrasound experiment for three different endpoints: chlorophyll-*a* concentration (Chl-*a*), photosystem II efficiency (Φ_PSII_) and particle concentration (# mL^−1^) of *Microcystis aeruginosa* PCC 7820. Homogeneous subgroups were defined by a Tukey *post hoc* comparison at *p* < 0.05.

Treatments	Chl-*a*(µg L^−1^)		Φ_PSII_ (-)		Particles (# mL^−1^)	
	Tukey post hoc comparison					
Control	a		a		a	
AL-05	a		b		b	
AL-10	a		b		b	
repeated measures ANOVA	*F*	*p*	*F*	*p*	*F*	*p*
treatment effect (*F*_2,6_)	3.84	0.084	64.5	<0.001	200.9	<0.001
time effect (*F*_5,30_)	423.3	<0.001	2.35	0.065	103.8	<0.001
time × treatment interaction (*F*_10,30_)	5.61	<0.001	1.56	0.169	42.9	<0.001

**Figure 6:**
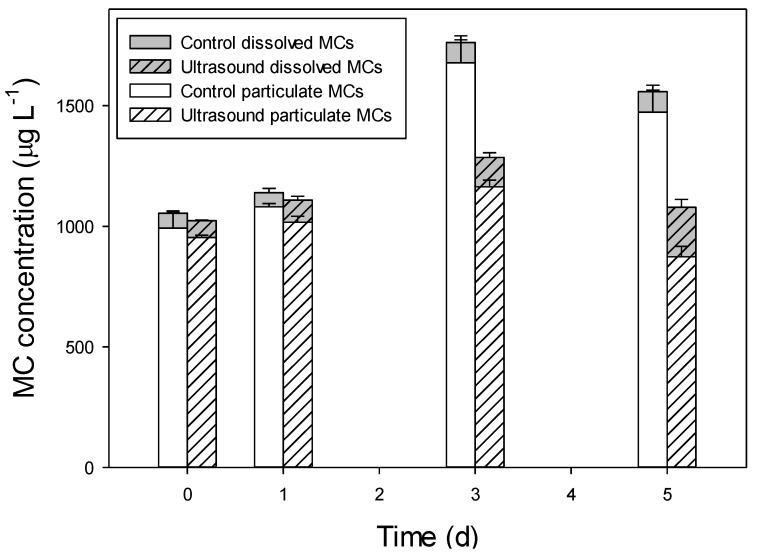
Particulate and dissolved microcystin (MC) concentrations (μg L^−1^) in non-exposed (open bars) and ultrasound (Flexidal AL-05)-exposed *Microcystis aeruginosa* PCC 7820 (hatched bars). Error bars indicate 1 SD (*n* = 3).

The effects of both transducers were similar; hence, only samples from the AL-05 treatments were analyzed for MCs. Total MCs significantly increased in the controls, from 1054 (SD 71) μg MC L^−1^ at the start to 1558 (SD 110) μg MC L^−1^ at the end of the experiment (*t*-test; *t* = 6.67; *p* = 0.003; Figure 6). The ultrasound treatment caused no decline in total MCs as total concentrations at the start (1023 SD 11 μg MC L^−1^) and after five days (1079 SD 34 μg MC L^−1^) were similar (*t* = 2.68; *p* = 0.055). The proportion of dissolved MCs after five days was significantly higher in the ultrasound treatments (19%) than in the controls (5%, *t* = 6.17; *p* = 0.004, Figure 6).

**Figure 7:**
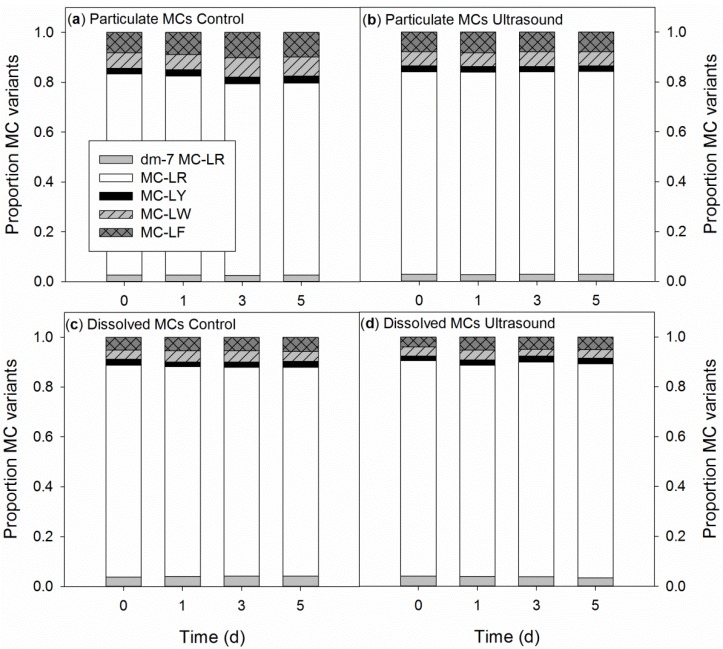
Course of the proportions of five microcystin (MC) variants in the particulate (**a**,**b**) and dissolved MC pools (**c**,**d**) of non-exposed *Microcystis aeruginosa* PCC 7820 populations (control) and populations exposed to ultrasound from Flexidal AL-05 transducers (ultrasound).

The composition of particulate MCs was similar during the course of the experiment in both controls and ultrasound treatments (Figure 7a,b) and resembled the particulate MC composition of the H_2_O_2_ experiment. Furthermore, the composition of dissolved MCs was similar in controls and the ultrasound treatments and remained stable during the experiment (Figure 7c,d). However, the composition of the dissolved MCs deviated slightly from the composition of particulate MCs (Figure 7). Two-way repeated-measures ANOVA revealed a significantly higher contribution of dm-7-MC-LR in the dissolved MCs (4%) than in the particulate MC pool (3%). Likewise, MC-LR was significantly more abundant in the dissolved pool (85%) than in the particulate MC pool (81%; *F*_1,8_ = 124.9; *p* < 0.001). MC-LY was present in similar proportions in both the dissolved and particulate MCs (2%; *F*_1,8_ = 4.0; *p* = 0.080), while MC-LW (*F*_1,8_ = 2311; *p* < 0.001) and MC-LF (*F*_1,8_ = 65.6; *p* < 0.001) were significantly less abundant in the dissolved MC pool (4% MC-LW and 5% MC-LF) than in the particulate MC pool, where they made up 6% (MC-LW) and 8% (MC-LF).

## 3. Discussion 

### 3.1. Hydrogen Peroxide

H_2_O_2_ effectively reduced *M. aeruginosa* biomass when dosed at 4 or 8 mg L^−1^, but not at 1 and 2 mg L^−1^. Hence, the EC_50_, depending on the endpoint, was between 2.5 and 3.8 mg H_2_O_2_ L^−1^ under our experimental conditions, which is a little higher than an EC_50_ of 0.3–0.5 mg L^−1^ reported for another strain of *M. aeruginosa* [31,32] and the dose of 2 mg H_2_O_2_ L^−1^ that was applied to a *Planktothrix agardhii*-dominated lake in The Netherlands, and removed 99% of the cyanobacteria [15]. However, in other field trials, 30%–40% of the cyanobacteria remained after an H_2_O_2_ treatment, despite the fact that dosages of 40 to 95 mg H_2_O_2_ L^−1^ were used [29,33]. For field collected colonial *M. aeruginosa*, a relatively high EC_50_ of about 30 mg H_2_O_2_ L^−1^ was found [34]. This high EC_50_ could have resulted from the use of extremely high cyanobacteria concentrations [34]. Alternatively, the mucilaginous colonies, often with quite a few bacteria attached [35], might provide *M. aeruginosa* cells protection against H_2_O_2_. Our strain of *M. aeruginosa* was uni- and bi-cellular; a normal growth form under laboratory conditions, but contrasting to its typical colonial appearance in the field [36]. Where an H_2_O_2_ concentration of 4 mg L^−1^ was sufficient to strongly reduce our strain, when blooms of colonial *Microcystis* are the target, higher concentrations may be needed. This might be a drawback in nature protection sites, as those H_2_O_2_ concentrations also may have an effect on non-target organisms, such as zooplankton [15,29,37].

The *M. aeruginosa* biomass reduction was probably a result of the loss of membrane integrity, followed by cell lysis [38,39]. Consequently, cell contents and cyanotoxins could be released upon treatment with H_2_O_2_. For instance, leakage of phycobiliproteins into the water after H_2_O_2_ exposure is reflected in Wang *et al.* [34], where a blue color at higher H_2_O_2_ dose(s) is clearly visible in their Figure 3. In our experiment, relatively high chlorophyll-*a* concentrations were present in the 8-mg L^−1^ H_2_O_2_ treatment after 24 h, while the particle concentration at that time had dropped to only 2.4% of the number of particles at the start of the experiment. Furthermore, after 24 h, dissolved MC concentrations in the 1-, 4- and 8-mg L^−1^ treatments had increased respectively 1.4-, 8.9- and 12-times compared to the control, which confirms compromised membrane integrity and leakage of cell contents. Similarly, lysis of cyanobacterial cells led to the appearance of dissolved MCs in the first few days after an H_2_O_2_ application in a waste water stabilization pond [29]. Some evidence for the release of MCs is also found in the field application of Matthijs *et al.* [15], where the MC decline was similar to the cyanobacterial biomass decline, but with a two-day delay. In contrast, a laboratory study using relatively high H_2_O_2_ concentrations (10.2–102 mg L^−1^) found no increase in dissolved MCs after the H_2_O_2_ treatments, which was attributed to the degradation of MCs [30]. In our experiment, we found indications that part of the MCs was also lost, as after 24 h, the sum of dissolved and particulate MCs in the 4- and 8-mg L^−1^ treatments were 23% lower than at the start of the experiment. As proposed by Song *et al.* [22], the degradation pathway is probably by hydroxyl radical attack on the Adda benzene ring, causing ring hydroxylation and subsequent oxidative cleavage of the Adda structure.

At the start, and after 24 h in the control and 1-mg L^−1^ treatments, the dissolved MCs contributed 6%–6.5% to the overall MC pool. This is in agreement with literature reports that most of the total detectable toxin pool is intracellular in healthy growing cells [40,41,42]. The intracellular MC composition in our study is in line with findings of others; MC-LR is the most abundant MC variant in *M. aeruginosa* PCC 7820, but its relative contribution might vary (Table 3). Variation in MC variant expression might be caused by different growth conditions (e.g., [43]). However, the difference between the intracellular and extracellular MC profiles in healthy cells observed in both of our experiments (Figure 4 and Figure 7) might point towards differences in terms of membrane crossing [44] or to different adsorption characteristics. We observed lower proportions of the more hydrophobic variants, MC-LW and MC-LF, in the dissolved fraction than in the particulate fraction of healthy cells. This difference can probably be attributed to a higher adsorption of the more hydrophilic variants on living cells. Although MCs do not easily adsorb on sediments or on suspended particulate matter [41], the more hydrophobic variants, MC-LW and MC-LF, were more associated with lipids in monolayer experiments than was the more hydrophilic MC-LR [45]. Adsorption of more hydrophobic MCs on remaining cells seems a more likely explanation than a passive diffusion mechanism [44], as that would result in a relatively higher proportion of hydrophobic MCs, as these diffuse more easily across the membrane. When most cells were lysed in the 4- and 8-mg H_2_O_2_ L^−1^ treatments, the proportions of the dissolved hydrophobic MCs increased (Figure 4d).

**Table 3:** The relative proportion (%) of different intracellular microcystin (MC) variants in *Microcystis aeruginosa* PCC 7820.

MC-variant	Our study	Ríos *et al.* [46]	Robillot *et al.* [44]
MC-LR	81.2	79.5	51.0
MC-LY	2.2	1.6	8.6
MC-LF	8.0	0.6	19.4
MC-LW	5.8	1.0	21.0
others	2.8	17.3	-

In the high H_2_O_2_ doses, total MC concentrations were about 23% reduced, but an H_2_O_2_ dose needed to oxidize all released MCs to levels below the WHO guideline of 1 μg L^−1^ would imply negative effects on non-target organisms, such as large bodied cladocerans. For example, LC_50_ values of 5–6 mg H_2_O_2_ L^−1^ for *Daphnia* and 2 mg L^−1^ for *Moina* have been reported [37]. Increased mortality for *Daphnia* was observed at H_2_O_2_ concentrations exceeding 3 mg L^−1^; for *Moina*, this occurred already at concentrations exceeding 1 mg H_2_O_2_ L^−1^ [29,37], while in enclosures, cladocerans were affected by H_2_O_2_ concentrations exceeding 2.5 mg L^−1^ [15]. In our study, more than 75% of the MCs remained present, even when a H_2_O_2_ dose of 8 mg L^−1^ was applied. This implies that algicides should be avoided as much as possible, because a sudden increase in dissolved MC concentrations might present a health hazard to livestock and humans using the water for consumption [11] and could potentially affect aquatic organisms that would not readily ingest cyanobacteria (e.g., [47]).

### 3.2. Ultrasound

Ultrasound can have detrimental effects on cyanobacteria; it can cause gas vesicle collapse, cell wall disruption and disturbance of photosynthetic activity [17,18]. Several commercial suppliers state that their “environmental-friendly” ultrasound transducers will kill cyanobacteria rapidly [48]. However, the low power used by these commercially available transducers, in contrast to the high power cavitation-producing transducers in some laboratory settings, puts strong constraints on their efficacy [19]. Indeed, our study confirmed the findings of Lürling and Tolman [19,49] that commercial ultrasound transducers do not remove cyanobacteria. In our experiment with *M. aeruginosa*, ultrasound somewhat impaired cyanobacterial growth, but the transducer was only placed in a small volume (800 mL). When applied in larger volumes, significantly less power will be transmitted, and consequently, the impact on cyanobacteria will be far lower when such transducers are employed in ponds and lakes [18], as recommended by the manufacturer [11]. Likewise, the marginal damage to the cyanobacterial cells, reflected by the small increase in dissolved MCs, will be less in larger volumes. In addition, possible direct effects of ultrasound on MC degradation will be less in large volumes, as MC degradation is proportional to the power and duration of ultrasound [21]. Intensities used to degrade MCs are much higher than the ones employed in our study. For instance, Song *et al.* [22] used 22.7 W mL^−1^ to degrade MCs, while the intensity used in our experiment was 7.9 × 10^−4^ W mL^−1^. High intensity ultrasound devices cause acoustic cavitation: a process in which compression and rarefaction create gas bubbles that may collapse [50]. On collapse of the bubbles, formation of radicals and hydrogen peroxide production may lead to degradation of MCs [22]. However, the power of the transducers applied in our study is probably insufficient to cause cavitation [19], and hence, strong ultrasound induced degradation of MCs was not observed; ultrasound caused no decline in total MCs, as total concentrations at the start of the experiment and after five days were similar.

Using high power devices to clear surface waters from cyanobacteria is not recommended, because the energy needed to treat lakes and ponds is very high, and non-target organisms will be damaged. For instance, ultrasound is used to disinfect ballast water or raw water for drinking water preparation, where it may inactivate motile plankton [51] or kill zooplankton, especially larger cladocerans [52]. Ultrasound caused cell damage in the macrophyte, *Elodea*, and in fruit flies [53]. Moreover, the commercial ultrasound transducers, as the ones we have used in our study, resulted in rapid death of the zooplankton grazer, *Daphnia* [49]. Therefore, ultrasound should not be considered “environmental friendly” [18] or a “green solution” [17] to control cyanobacteria blooms.

Some studies have reported ultrasound-induced cyanobacterial filament shortening by breakage and cell lysis at the break points [19,54]. This could lead to the release of toxins in the water. Our strain of *M. aeruginosa* was completely uni- and bi-cellular. However, in the field, *M. aeruginosa* grows mostly as large colonies, embedded in mucous. There is a possibility that application of commercial ultrasound devices could break natural colonies into smaller fragments, but no major impact, such as on toxin release, is expected.

The composition of the MC pools was not influenced by the ultrasound and the MC profiles remained similar during the experiment. The only difference was between the composition of the dissolved MC pool and the particulate MC pool that showed less hydrophobic MC-LW and MC-LF in the dissolved pool than in the particulate one, as had also been observed in the H_2_O_2_ experiment.

### 3.3. Overall

In this study, we simulated heavy bloom concentrations of *M. aeruginosa* (≈700 μg chlorophyll-*a* L^−1^) and treated them with H_2_O_2_ and ultrasound from commercial transducers. Where doses of 4 to 8 mg H_2_O_2_ L^−1^ were sufficient to kill *M. aeruginosa*, MC concentrations in the water remained high, and the majority of the particulate MCs became dissolved. The high cyanobacteria concentrations we used are not uncommon in urban ponds in The Netherlands [55], or elsewhere (e.g., [56,57,58,59]), and even higher concentrations are common in surface scums in ponds and lakes. Treating such blooms (and scums) with H_2_O_2_ may only be feasible if *P. agardhii* is the dominant species [60], but with *M. aeruginosa* dominating, it is not recommended, as the relatively high H_2_O_2_ concentrations needed for an effective treatment might harm non-target organisms and might cause a release of cell contents, including toxins and nutrients, which may fuel a subsequent bloom. Moreover, as previously reported [29,34], H_2_O_2_ treatment may result in surface accumulation of affected cyanobacterial cells. Such accumulation of decaying material will in itself cause nuisance and reduce water quality.

Ultrasound from the commercial available transducers was not effective at clearing a small volume of 800 mL of a cyanobacterial suspension. Hence, these devices will not be effective in larger volumes, which is in line with additional laboratory experiments [19], a tank experiment [49] and a few lake-scale experiments where ultrasound appeared ineffective at mitigating cyanobacteria nuisance [61,62]. Consequently, the application of ultrasound is not a suitable way to reduce cyanobacterial biomass. 

Water authorities should perform a system analysis to determine the flow of water and sources of nutrients in systems suffering from blooms, after which the most promising set of mitigating measures can be selected. Here, source-oriented measures principally targeting the phosphorus inflow and internal loading remain essential for reducing eutrophication. However, in situations where source-oriented measures are not easily achievable, cost-effective end-of-pipe solutions might be an alternative, but these should have been proven to be effective *in situ*. For instance, coagulants in combination with a ballast can effectively precipitate positively buoyant cyanobacteria out of the water column as intact cells [63,64] without releasing cell contents and, thereby, nutrients and toxins [65,66].

## 4. Experimental Section 

### 4.1. Organisms

The cyanobacterium, *Microcystis aeruginosa* (Kützing) Kützing strain PCC 7820, was obtained from the Pasteur Culture Collection (Paris, France). The culture was maintained in 250-mL Erlenmeyer flasks that contained 100 mL sterile modified WC (Woods Hole modified CHU10) medium [67] and were closed with a cellulose stopper. The flasks were placed at 25 °C in 40 µmol quanta m^−2^ s^−1^ provided in a 14:10 h light-dark cycle. Stock cultures were transferred to fresh medium every two to three weeks. These stocks were used to inoculate twenty 2-L Erlenmeyer flasks with 1.7 L sterile WC-medium to produce sufficient biomass for the experiments. The 2-L flasks were placed in a conditioned climate room at 23 °C in a 12:12 h light dark cycle at a light intensity of 100 µmol quanta m^−2^ s^−1^. The flasks were shaken manually once a day. Regularly, a 2-mL subsample was taken for chlorophyll-*a* determination by a PHYTO-PAM phytoplankton analyzer (Heinz Walz GmbH, Effeltrich, Germany). Experiments were started with the cultured material once the chlorophyll-*a* concentration was 600–800 μg L^−1^ (equivalent to cell concentrations of on average 3.8 × 10^6^ cells mL^−1^ SD 0.3 × 10^6^ cells mL^−1^). Hereto, aliquots of 800 mL cultured *M. aeruginosa* were transferred from the 2-L flasks to experimental glass jars (1 L volume). The remaining cultures in the 2-L Erlenmeyer’s were replenished with fresh, sterile WC-medium and placed back for further culturing. The experiments were conducted at 23 °C in 100 µmol quanta m^−2^ s^−1^ light intensity with 12:12 h light-dark cycle in a conditioned climate room.

### 4.2. Hydrogen Peroxide

At the start of the experiment, suspensions of *M. aeruginosa* in WC-medium were evenly distributed over 15 replicate 1-L jars such that each contained 800 mL suspension with a chlorophyll-*a* concentration of 702 (SD 23) µg L^−1^. Hydrogen peroxide (H_2_O_2_ 30%, 1.07209.0500, Merck KGaA, Darmstadt, Germany) was added in triplicate to the jars in concentrations of 0, 1, 2, 4 and 8 mg L^−1^. H_2_O_2_ was gently added under continuous stirring. The jars were sealed with Parafilm with a small opening for ventilation and sampling. At 0, 1, 4 and 24 h, MC samples (8 mL, stored in glass bottles) and samples for chlorophyll-*a* and cell density measurements (10 mL) were taken from the middle of the jar after mixing with a glass stick. Cyanobacterial chlorophyll-*a* concentrations and photosystem II efficiency were measured directly after sampling by a PHYTO-PAM phytoplankton analyzer; particle concentrations were measured by an electronic particle counter (CASY cell counter, Schärfe System GmBh, Reutlingen, Germany) with a range from 1 to 120 μm equivalent spherical diameter. MCs were determined as outlined below (Section 4.4).

### 4.3. Ultrasound

Seven ultrasound devices (three of type AL-05 and four of type AL-10, Flexidal BVBA, Aalter, Belgium) were purchased commercially. All ultrasound transducers produced block waves at frequencies of ~20 kHz, ~28 kHz and ~44 kHz. The acoustic power of the transducers was 0.63 W (SD 0.05, *n* = 3) for AL-05 transducers [49] and 0.68 W (SD 0.23, *n* = 4) for the AL-10 transducers [19].

At the start of the experiment, suspensions of *M. aeruginosa* in WC-medium were evenly distributed over 9 replicate 1-L jars, such that each contained an 800-mL suspension with a chlorophyll-*a* concentration of 649 (SD 24) µg L^−1^. In three jars, AL-05 transducers were placed 2 cm below the water surface; in three other jars, AL-10 transducers were placed at a similar depth; while three jars remained untreated (controls). The ultrasound transducers operated incessantly during the experiment for 5 days. Sub-samples of 10 mL for chlorophyll-*a*, photosystem II efficiency and cell density analysis (electronic particle counter) were taken daily during the experiment (Days 0 to 5) after mixing with a glass stick. MC samples (8 mL stored in glass bottles) were taken from the water column after mixing at 0, 1, 3 and 5 days. MCs were determined as outlined below (Section 4.4).

### 4.4. MC Analysis

The 8-mL MC samples were filtered through glass-fiber filters (Whatman GF/C, Whatman International Ltd., Maidstone, UK). Of each sample, the filtrate was collected in clean 8-mL glass tubes, while the filters were rolled gently and placed in another 8-mL glass tube. Both the filtrate and the filter samples were dried in a Speedvac (Thermo Scientific Savant SPD121P, Waltham, MA, USA), after which the filters were extracted three times at 60 °C in 2.5 mL 75% methanol-25% Millipore water (*v*/*v*). The extracts were dried in the Speedvac and subsequently reconstituted in 800 μL methanol, as were the dried filtrates. The reconstituted samples were transferred to 2-mL Eppendorf vials with a cellulose-acetate filter (0.2 μm, Grace Davison Discovery Sciences, Deerfield, IL, USA) and centrifuged for 5 min at 16,000× *g* (VWR Galaxy 16DH, VWR International, Buffalo Grove, IL, USA). Filtrates were transferred to amber glass vials for LC-MS/MS analysis.

Samples were analyzed for eight MC variants (dm-7-MC-RR, MC-RR, MC-YR, dm-7-MC-LR, MC-LR, MC-LY, MC-LW and MC-LF) and nodularin (NOD) by LC-MS/MS, as described in [68]. LC-MS/MS analysis was performed on an Agilent 1200 LC and an Agilent 6410A QQQ. The compounds were separated on an Agilent Eclipse XDB-C18 4.6 × 150 mm, 5-μm column by Millipore water with 0.1% formic acid (*v*/*v*, Eluent A) and acetonitrile with 0.1% formic acid (*v*/*v*, Eluent B). The elution program was 0–2 min 30% B, 6–12 min 90% B, with a linear increase of B between 2 and 6 min and a 5-min post run at 30% B. The injection volume was 10 μL; flow was 0.5 mL/min; column temperature was 40 °C. The LC-MS/MS was operated in positive mode with an electrospray ionization source, and nitrogen was used as the drying and collision gas. For each compound, two transitions were monitored in Multiple Reaction Monitoring (MRM) mode. The first quadrupole was operated in unit mode; the second quadrupole was operated in the widest mode. The dwell time was 50 ms. MS/MS settings for each compound were as in Faassen and Lurling [68]. Calibration standards were obtained from DHI LAB Products (Hørsholm, Denmark) and prepared in methanol. Samples were quantified against a calibration curve, and filter extracts were subsequently corrected for recovery. Each sample was injected once. Information on recovery, repeatability, limit of detection and limit of quantification of the analysis is given in [68].

### 4.5. Statistical Analysis

The chlorophyll-*a* concentrations, photosystem II efficiencies and particle concentrations were statistically evaluated by repeated measure ANOVAs in the tool pack SPSS (version 19.0, IBM statistics, Armonk, NY, USA). Homogeneous subgroups were defined by a Tukey *post hoc* comparison at *p* < 0.05. The data were checked for normality using quantile-quantile (Q-Q) plots. In case Mauchly’s test indicated that the assumption of sphericity had been violated, the degrees of freedom were corrected using Greenhouse-Geisser estimates of sphericity if epsilon <0.75 or applying the Huynh-Feldt correction if epsilon >0.75.

Total MC concentrations were evaluated statistically running one-way ANOVAs in the peroxide experiment and by *t-*ests in the ultrasound experiment using the toolpack, SigmaPlot version 12.3 (Systat Software, Inc., San Jose, CA, USA). The composition of both the dissolved and the particulate MC pools at the start and at the end of the peroxide experiment was evaluated by two-way ANOVAs in SigmaPlot with dissolved/particulate and peroxide concentration as fixed factors. Data were checked for normality and heteroscedasticity by the normality test (Shapiro–Wilk) and the equal variance test in SigmaPlot prior to executing ANOVA. The ANOVAs were followed by pairwise multiple comparison procedures (Holm–Sidak method) to distinguish means that were significantly different (*p* < 0.05). In the ultrasound experiment, the relative contribution of each MC variant in the dissolved and particulate MC pools in controls and ultrasound exposures was evaluated over time running two-way repeated measures ANOVAs in SPSS (version 19.0, IBM statistics, Armonk, NY, USA).

## 5. Conclusions

Hydrogen peroxide at concentrations of 4 and 8 mg L^−1^ effectively killed *M. aeruginosa*, but caused substantial release of MCs into the water. About 23% of the total MCs was removed by these hydrogen peroxide doses. To eliminate most MCs from the water, much higher H_2_O_2_ concentrations will be needed, which might strongly impair non-targeted organisms, such as the grazer, *Daphnia magna*.

The application of ultrasound had no water clearing effect; it caused a minimal growth inhibition and some release of MCs into the water. Ultrasound from commercial transducers is ineffective at controlling cyanobacteria in surface waters.

The MC composition of the particulate and the dissolved fraction differed. The more hydrophobic variants, MC-LW and MC-LF, were underrepresented in the dissolved fraction of healthy cells. When cells were lysed, more hydrophobic variants appeared in the dissolved pool, but their abundance was still lower than in the particulate pool.
